# Fungal Endophyte Diversity and Bioactivity in the Indian Medicinal Plant *Ocimum sanctum* Linn

**DOI:** 10.1371/journal.pone.0141444

**Published:** 2015-11-03

**Authors:** Kanika Chowdhary, Nutan Kaushik

**Affiliations:** 1 TERI University, 10^th^ Institutional Area, Vasant Kunj, New Delhi, India; 2 The Energy and Resources Institute (TERI), India Habitat Center, Lodhi Road, New Delhi, India; Bhabha Atomic Research Centre, INDIA

## Abstract

Endophytic mycopopulation isolated from India’s Queen of herbs Tulsi (*Ocimum sanctum*) were explored and investigated for their diversity and antiphytopathogenic activity against widespread plant pathogens *Botrytis cinerea*, *Sclerotinia sclerotiorum*, *Rhizoctonia solani* and *Fusarium oxysporum*. 90 fungal isolates, representing 17 genera were recovered from 313 disease-free and surface sterilised plant segments (leaf and stem tissues) from three different geographic locations (Delhi, Hyderabad and Mukteshwar) during distinct sampling times in consequent years 2010 and 2011 in India. Fungal endophytes were subjected to molecular identification based on rDNA ITS sequence analysis. Plant pathogens such as *F*. *verticillioides*, *B*. *maydis*, *C*. *coarctatum*, *R*. *bataticola*, *Hypoxylon sp*., *Diaporthe phaseolorum*, *Alternaria tenuissima and A*. *alternata* have occurred as endophyte only during second sampling (second sampling in 2011) in the present study. Bi-plot generated by principal component analysis suggested tissue specificity of certain fungal endophytes. Dendrogram revealed species abundance as a function of mean temperature of the location at the time of sampling. Shannon diversity in the first collection is highest in Hyderabad leaf tissues (H' = 1.907) whereas in second collection it was highest from leaf tissues of Delhi (H' = 1.846). Mukteshwar (altitude: 7500 feet) reported least isolation rate in second collection. Nearly 23% of the total fungal isolates were considered as potent biocontrol agent. Hexane extract of *M*. *phaseolina* recovered from Hyderabad in first collection demonstrated highest activity against *S*. *sclerotiorum* with IC_50_ value of 0.38 mg/ml. Additionally, its components 2H-pyran-2-one, 5,6-dihydro-6-pentyl and palmitic acid, methyl ester as reported by GC-MS Chromatogram upon evaluation for their antiphytopathogenic activity exhibited IC_50_ value of 1.002 and 0.662 against respectively *S*. *sclerotiorum* indicating their significant role in antiphytopathogenic activity of hexane extract. The production of 2H-pyran-2-one, 5,6-dihydro-6-pentyl from *M*. *phaseolina*, an endophytic fungus is being reported for the first time.

## Introduction

Endophytic fungi inhabit a unique biological niche and are categorized as highly diverse, polyphyletic group of primarily ascomycetous fungi, capable of colonizing tissues of plants asymptomatically without initiating any disease or overt negative symptoms [1], [2], [3]. Endophytes have been recovered from all plants explored for their presence till date [4], [5]. 420,000 plant species exist in nature and only a few have been completely studied relative to their endophytic biology [6]. Diversity of fungal endophytes is 7% out of total of 1.5 million fungi on earth [7], [8]. Fungal endophytes are believed to be a treasure of structurally and biologically unique natural products as documented previously in several reviews [9], [10], [11], [12], [13], [14]. Numerous antifungal metabolites have been reported from endophytic fungi belonging to different structural classes such as alkaloids, peptides, steroids, terpenoids, phenols, quinines and flavonoids [15].

*Ocimum sanctum* is ubiquitous in Indian culture and tradition, called *Tulsi* in Hindi[16]. Because of its well documented therapeutic potential, Ayurveda (Indigenous system of Indian medicine) describes *O*. *sanctum* as *Sashemani Shwasaharani* (antiasthmatic), *Kaphaghna* (suppressant drug) and believed to promote longevity [17], [18], [19]. It is mentioned as adaptogenic in nature, balancing different processes in the body and helpful for adopting to stress in *Charaka Samhita* [20].

Besides, it’s numerous pharmacological benefits, *Ocimum sanctum* extracts and essential oils particularly eugenol have been known to be highly effective against a plethora of plant pathogens. Essential oils extracted from Uttarakand grown *Ocimum sanctum* displayed antifungal activity against *Rhizoctonia solani* with MIC of 62.5 μg/ml [21]. *O*. *sanctum* plant extracts have also shown 38.15% inhibition against *Sclerotinia sclerotiorum*de Bary causing stem rot of Indian mustard [22]. Similarily leaves of *Ocimum sanctum* has been reportedly found effective in inhibition of alfatoxin producing fungi *Aspergillus parasiticus* by 7–20% [23].

Therefore, the objective of the work reported in this manuscript was to do systematic study of diversity and distribution of endophytic fungi isolated from different tissues of *Ocimum sanctum* collected from three different geographic locations and in two variable sampling times (seasons), and further screen them as potential biocontrol agents against four broad spectrum plant fungal pathogens and finally to scrutinize their metabolite profile with GC-MS Chromatography.

## Materials and Methods

### Collection of host plants

In order to maximize the chances of getting bioactive endophytic fungi, collection of host plants was done in different agroclimatic zones varying widely in climatic conditions, namely Southern plateau and hills region: Hyderabad (17.3660°N, 78.4760°E); Western Himalayan region: Mukteshwar (29.4722°N, 79.6479° E) and Trans Gangetic plains: Delhi and Gual pahari (28.4700°N,77.0300°E) in two different sampling time (autumn: (August-September) and summer (April-June) between two consequent years 2010 and 2011 with recorded variations in mean temperature °C and rainfall as mentioned in Table 1. Host plant samples were collected from healthy, disease free cultivated plant hosts from the above mentioned places for isolation of endophytic fungi. All the individuals of medicinal host plant *Ocimum sanctum* were collected from cultivated herbal gardens in all the locations. No particular variety of *O*. *sanctum* was selected. Same individuals were investigated in different seasons/sampling times. Endophytic fungi were isolated only from leaf and stem parts. Plants were freshly transported in moist ziplocked polybags from collection site to laboratory in Delhi. Plant parts (leaf and stem) were thoroughly washed in running tap water and processed for isolation of endophytic fungi immediately (within 1–2 days) after collection of whole plant.

**Table 1:** Characteristics and conditions of sites.

**Geographic Locations**	Hyderabad	Mukteshwar	Delhi and Gual Pahari
**Institutes**	Herbal Garden at ANGR agricultural University	Herbal Garden at TRISHA, Supi	Herbal Garden TERI Gram, Gual pahari
**Coordinates** ^a^	17.3660°N, 78.4760°E	29.4722°N, 79.6479° E	28.4700°N,77.0300°E
**Mean rainfall (mm)2011** ^b^	September-240.9	August-702.5	September- 359.7
**Mean rainfall (mm)2011** ^b^	June-34.8	June-334.2	April-23.0
**Mean temperature °C 2010** ^b^	September-24°C	August-14°C	September- 31°C
**Mean temperature °C 2011** ^b^	June-30°C	June-17.7°C	April-23.0°C

^a^(http://www.agriinfo.in);

^b^ (source: http://www.imd.gov.in).

#### Ethics statement

No specific permissions were required for collection of host plants from above stated locations. At Mukteshwar and Delhi, *O*.*sanctum* plants were procured by first author Ms. Kanika Chowdhary from Host Institution’s (The Energy and Resources Institute) Herbal Garden, being in the capacity of its Research student. Whereas in Hyderabad *O*.*sanctum* plants were procured from Herbal Garden of a regional Agricultural University and specific permission was not required as they were available for sale for public. (Cash bill from Herbal Garden, Hyderabad is appended as S1 Fig). It is hereby confirmed that field studies didn’t involve endangered or protected species.

#### Data availability

All relevant data are within the paper and its supporting information files. Also, minimal dataset needed for replication is available within the manuscript, supporting information and stored in a stable repository as well.

### Isolation of endophytic fungi and maintenance

Surface Sterilization for isolation of endophytic fungi was done following previously established procedures [24]. Washed plant parts were treated by the following immersion sequence: 70% ethanol for 2 mins followed by 1% sodium hypochlorite (NaOCl) solution for 3 mins. Thereupon, samples were rinsed in double distilled, sterilized water for a couple of minutes. Then samples were dried on a blotting sheet. Imprints of dried and sterilized samples were taken on media plates; finally samples were chopped into 8 mm diameter segments and placed (3–4 segments on each plate) onto petri dishes (Tarsons, Kolkata) containing malt agar as medium. Malt extract-Agar medium {Malt extract (Himedia, Mumbai)15g, Agar powder (Himedia, Mumbai)15 g dissolved in 1000ml distilled water} amended with antibiotics chloramphenicol (Himedia, Mumbai) @ 0.2g/l and streptomycin sulphate (Himedia, Mumbai) @ 0.1 g/l of media at 7.4–7.8 pH was used as an medium for isolation, purification and maintenance of endophytic fungi. The plates were incubated at 24 ± 2°C for upto three weeks. The plant segments were observed once a day for the growth of the endophytic fungi. Hyphal tips of the endophytic fungi growing from the plant segments were isolated from isolation plate and maintained on fresh malt agar plates and coded with a unique number until identified.

### Total genomic DNA extraction, PCR amplification and sequencing

Total genomic DNA of the endophytic fungi was isolated directly from freshly subcultured endophytic fungi grown on Malt extract-agar medium, using the DNeasy plant minikit (Qiagen), according to manufacturers’ protocol. PCR amplification of isolated DNA was carried out by performing slight modification in previously described method [24]. The universal primers ITS1 5′ TCCGTAGGTGAACCTGCGG 3′ and ITS4 5′ TCCTCCGCTTATTGATATGC 3′ (Sigma Aldrich) were used to amplify the ITS region in rDNA. PCR mixture (50 μl) for each sample consisted of 2 μl (100 ng/μl) of DNA template, 1 μl (10pmol/μl) of each primer, 0.4 μl Taq polymerase, 1 μl dNTPs, PCR buffer (10×) 5 μl and 39.6 μl MQ water (Applied Biosystems). The PCR reactions were performed in thermal cycler (Applied Biosystems) with the following conditions: Initial denaturation at 95°C for 15 min, 35 cycles at 95°C (denaturation) for 1 min, 56°C (annealing) for 30 secs, 72°C (extension) for 1 min and then a final extension for 10 min at 72°C. PCR products were sent to Eurofins laboratories Bangalore (India) for sequencing.

### Identification of endophytic fungi and phylogenetic evaluation

To identify the isolates, sequences were subjected to the BLAST search with the NCBI database. Multiple sequence alignment of approximately 500 bp sequences was performed using CLUSTAL W version 1.8. The phylogenetic tree was reconstructed and the evolutionary history inferred using the Neighbor-Joining method [25]. Tree topologies were evaluated by performing bootstrap analysis of 1,000 dataset [26] with the updated MEGA 6 [27], [28]. The sequences of this study were deposited at GenBank. The accession numbers are detailed in Table 2.

**Table 2:** Molecular identification of fungal endophytes recovered from *O*. *sanctum* based on ITS rDNA analysis.

Endophytic fungi isolated (BLAST Search Results)/GenBank accession number	No of Isolates	Family to which endophytic fungus belongs	% Similarity of the sequence	% Query coverage	No. of basepairs sequencedand analyzed	Relative abundance
*Alternaria alternata* (KR017024)	5	Pleosporaceae	97%	95%	547	5.56%
*Alternaria sp*. (KR017025)	3	Pleosporaceae	96%	86%	592	3.33%
*Alternaria tenuissima* (KT428769)	3	Pleosporaceae	96%	86%	592	3.33%
*Aspergillus niger* (KR017026)	9	Trichocomaceae	79%	72%	550	10%
*Bipolaris maydis* (KR017029)	2	Pleosporaceae	92%	90%	500	2.2%
*Chaetomium coarctatum* (KR017030)	6	Chaetomiaceae	91%	95%	557	6.67%
*Colletotrichum sp*. (KR017032)	4	Glomerellaceae	100%	99%	368	4.4%
*Curvularia lunata* (KR017033)	1	Pleosporaceae	82%	96%	573	1.1%
*Diaporthe phaseolorum*(KR017034)	7	Diaporthaceae	99%	99%	495	7.8%
*Fusarium proliferatum* (KR017049)	6	Nectriaceae	97%	93%	515	6.67%
*Fusarium solani* (KR017036)	2	Nectriaceae	99%	99%	519	2.2%
*Fusarium verticillioides* (KR017037)	1	Nectriaceae	99%	96%	520	1.1%
*Hypocrea sp*. (KR017038)	7	Hypocreaceae	99%	95%	604	7.8%
*Hypoxylon sp*. (KR017039)	3	Xylariaceae	99%	99%	467	3.33%
*Macrophomina phaseolina* (KR017040)	7	Botryosphaeriaceae	97%	96%	565	7.8%
*Meyerozyma guilliermondii* (KR017041)	2	Debaryomycetaceae	87%	72%	591	2.2%
*Meyerozyma sp*. (KR017042)	1	Debaryomycetaceae	97%	98%	534	1.1%
*Penicillium crustosum* (KR017043)	1	Trichocomaceae	96%	96%	614	1.1%
*Penicillium sp*. (KR017044)	5	Trichocomaceae	98%	97%	546	5.56%
*Rhizoctonia bataticola* (KR017045)	6	Botryosphaeriaceae	91%	92%	534	6.67%
*Rhizopus oryzae* (KR017046)	4	Mucoraceae	97%`	98%	495	4.4%
*Setosphaeria rostrata*(KR017047)	3	Pleosporaceae	96%	93%	564	3.33%
*Sympodiomyces sp* (KR017048)	2	Trichomonascaceae	90%	64%	599	2.2%

### Dual culture bioassay

After purification of each endophytic fungal species, dual culture bioassay was conducted against economically significant plant pathogenic fungi e.g. *Rhizoctonia solani*, *Sclerotinia sclerotiorum*, *Fusarium oxysporum*, *Botrytis cinerea*; to test the antiphytopathogenic activity of the given endophyte fungi. Potato dextrose agar medium (Fresh Potato 15gms, distilled water 1000ml, D-glucose 15gms and Agar powder 15gms) was selected for dual culture as it favours growth of plant pathogenic fungi. The pathogen cultures were obtained from Indian Type Culture Collection, Indian Agricultural Research Institute, New Delhi. Both endophytic fungi (under investigation) and plant pathogenic fungi were placed with the aid of cork borer (8mm diameter) on PDA plate respectively opposite to each other and left for incubation at 24±2°C. After 6–7 days of incubation plates were observed and antagonism expressed and the various modes of interactions possible between endophytic fungi and pathogenic fungi were duly recorded. The experiments were conducted with 3 set of replication plates.

### Multiplication of selected endophytic fungal isolates

Thirteen endophytic fungal isolates showing antagonistic activity at least with 3 pathogenic fungi were further explored for antiphytopathogenic activity of their crude extracts by multiplication on rice media. Rice medium was prepared in 1L of Erlenmeyer flasks containing 300gms of Rice grain (autoclaved) [29]. The flasks were inoculated with 1-2ml distilled water suspension of hyphae scrapped from freshly cultivated culture of potential fungal endophyte under static conditions in daylight at 25±2°C.

### Chemical extraction of crude extracts of endophytic fungal isolates

200 ml of ethyl acetate (Qualigens, India) was poured while stirring on the rice grains covered with profuse growth of potential fungi and kept aside for 24 hrs. After which the contents were filtered under vacuum using a buchner funnel and extraction was repeated twice with ethylacetate for complete extraction of metabolites. The pooled extract was then dried up with vacuum rotary evaporator (Heidolph, Germany) under reduced pressure at 40°C which served as crude extract for further evaluation [29].

### Preliminary phytochemical analysis of crude fungal extract for presence of terpenoids

Since plant parts of *O*. *sanctum* are reservoir of essential oils, fungal endophytes isolated from them were investigated for presence of terpenoids which are primary constituents of essential oils. To the crude extract chloroform (Qualigens, India) and conc. H_2_SO_4_ (Rankem, India) were added to form a lower layer [30], [31]. Formation of reddish brown color confirms the presence of terpenoids. Fungal crude extracts showing presence of terpenoids were further subjected to partitioning between n-hexane (Rankem, India) and 90% methanol (Qualigens, India). Confirmation of presence of terpenoids in hexane extracts was done by GC-MS Chromatography.

### Gas chromatography mass spectroscopy (GC-MS)analysis of hexane extract

The GC-MS analysis of hexane extracts was carried on GC-MS (Agilent Technologies 7890A). For this 1mg of extract was dissolved 1 ml LR-grade dichloromethane (Qualigens, India). DB-WAX column (30 m × 250μm × 0.25 μm) was used with helium as a carrier gas at a flow rate of 1 ml/min at pressure of 11.654 psi. The GC oven temperature was kept at 120°C for 2 min and programmed to 5°C/min to 130°C for 1 min; 5°C/min to 150°C for 2 min; 2°C/min to 180°C for 3 min; 2°C/min to 200°C for 3 min; 5°C/min to 240°C for 20 min. Splitless injections were done with liquid injection method in this study. A library search was carried out using NIST library.

### Poisoned food bioassay of endophyte fungal crude extract and pure compounds

Poisoned food bioassay of endophytic crude extracts was conducted by means of biometric agar dilution method [32]. Dried crude extract/pure compound was dissolved in methanol (Stock solution: 40mg/ml). From the stock solution 125, 62.5, 12.5,6.25 and 1.25 μl were dissolved in 5ml of PDA medium to obtain five different concentrations i.e. 1mg/ml, 0.5mg/ml, 0.1mg/ml, 0.05 mg/ml and 0.01 mg/ml respectively. Intoxicated media plates were inoculated with 8 cork borer plugs of plant pathogenic fungi measuring 3mm^2^ placed in such a way that seven are put in periphery and one in the centre of the plate. The control experiment consisted of only methanol solvent in PDA medium [33]. % GI was calculated for each set of bioassay using following formula:

GI= {(A−B)/A} x 100

Where A = radial diameter of plant pathogenic fungi in check plate with methanol solvent only.

B = radial diameter of plant pathogenic fungi in extract /fraction intoxicated plates

IC_50_ was calculated by regression equation analysis. For calculating IC_50_ value, %GI at five different concentrations (mg/ml) were put in excel 2010 and scatter plot was generated. Linear trendline was produced on it displaying regression equation. Finally, mathematically (where, Y = 50) solving the equation provides the IC_50_ value.

### Data analysis

#### Isolation rate

Isolation rate was calculated as the number of isolates obtained from segments/pieces divided by the total number of segments/pieces [34].

#### Relative frequency

Relative frequency was calculated as the number of isolates of one species divided by the total number of isolates, and expressed as percentage [34].

#### Menhinick’s index

Species richness among the isolated endophytic fungi was determined by calculating the Menhinick’s index (Dmn) using the following equation [35]:

Dmn=s/√N

where, s is the number of different endophytic species in a sample (plant tissue) and N is the total number of isolated endophytic fungi in a given sample.

#### Camargo’s index

The fungal dominance can be determined by Camargo’s index (1/D_*mn*_), where D_*mn*_ represents species richness.

#### Shannon diversity index

Furthermore, to quantify the endophytic fungal diversity the Shannon diversity index (H′) was calculated using the following equation:

$$\text{H}\prime =-\underset{i}{\sum }Pi\text{ln}\left(Pi\right)$$

Where, Pi is relative abundance of a species is in a given sample H′ values could start from 0 (only one species present with no uncertainty as to what species each individual will be) and go higher revealing high uncertainty as species are relatively evenly distributed.

#### Plieou’s evenness index

J′=H′/ln(S)

where, H′ is the Shannon diversity of the endophytic fungi in a given sample and S is the total number of endophytic fungal individuals present in the given sample [36], [37], [38], [39].

The similarity and of endophytic fungal assemblages among both tissues was compared using the following similarity indices:

#### Sorensen’s index of similarity

(QS), QS = 2a/(2a + b + c) where ‘a’ is the number of common species in both communities, while ‘b’ and ‘c’ are the number of species specified to each community under investigation, respectively [40].

#### Jaccard’s index of similarity

JS was calculated using the formula: JS = a/(a + b + c) where ‘a’ is the number of common species in both communities, while ‘b’ and ‘c’ are the number of species specified to each community, respectively [41].

#### Principal component and cluster analysis

Principal component analysis studied interrelationships between endophytic fungi recovered from *O*. *sanctum* different plants parts in distinct sampling time, whilst cluster analysis deduced concurrence between species richness and temperature of geographical locations at the time of sampling. For this, software Unsrcambler X: version 10, CAMO, USA was used [42].

## Results

### Identification of fungal endophytes

**Fig 1:**
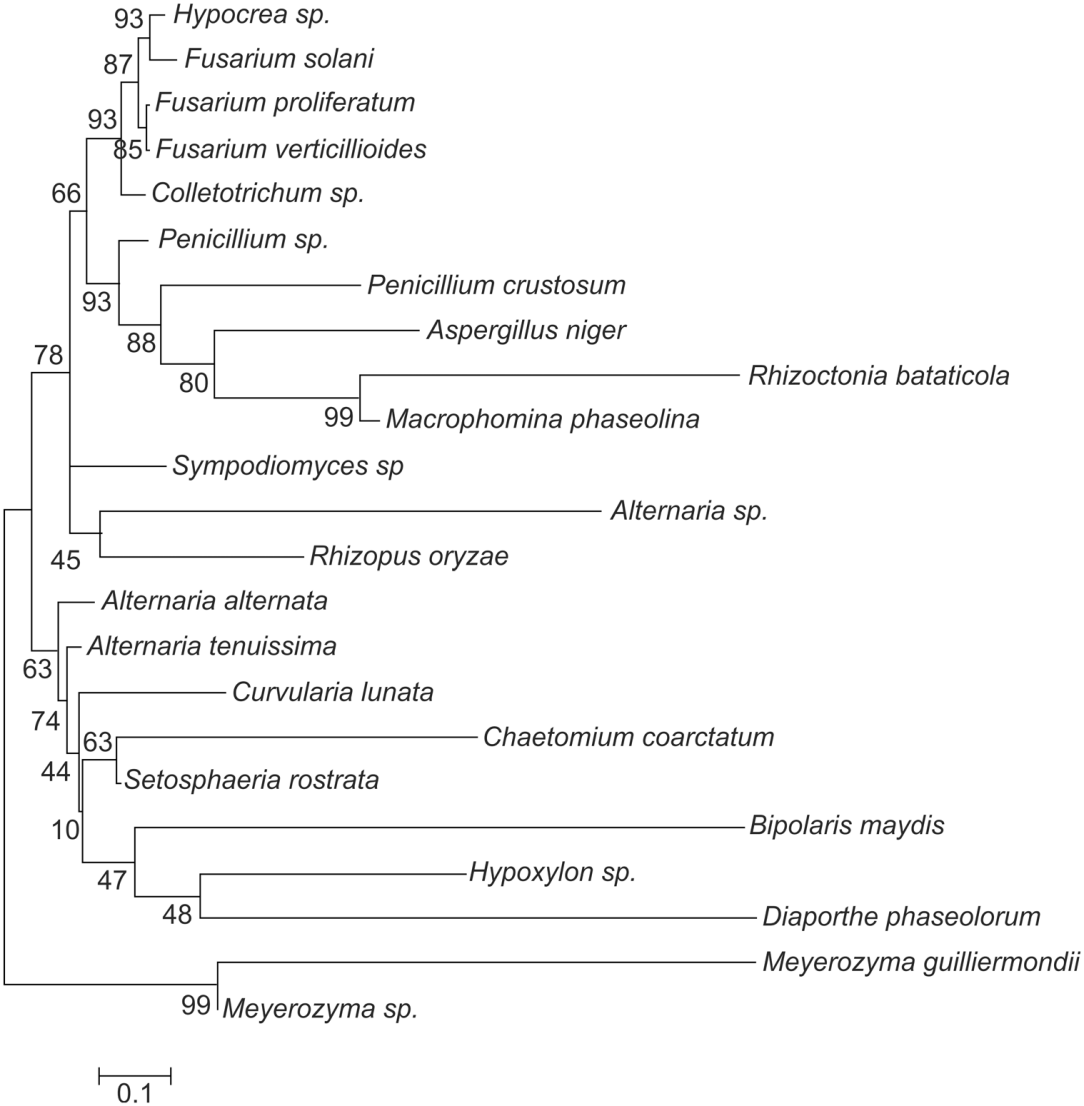
Phylogenetic tree based on neighbor-joining analysis of the rDNA ITS sequences of the endophytic fungal isolates obtained from various tissues of *Ocimum sanctum*. The bootstrap consensus tree inferred from 1,000 replicates.

90 endophytic fungi were isolated based on culture dependent technique. Fungal isolates were grouped into 43 different morphospecies according to their morphological characteristics: colony colour and texture, border type, and radial growth rate on MA media. All isolates of each morphospecies group were submitted to molecular identification based on rDNA ITS sequence analysis. Based on the molecular identification these morphospecies were grouped into 23 species. The detailed description of 23 different endophytic fungal isolates with respective identification, accession number from GenBank, % similarity, % query coverage and number of base pairs sequence analysed are summarised in Table 2. The evolutionary history was inferred using the Neighbor-Joining method (Fig 1). The optimal tree with the sum of branch length = 72.08408877 is shown. The percentage of replicate trees in which the associated taxa clustered together in the bootstrap test (1000 replicates) are shown next to the branches. The tree is drawn to scale, with branch lengths in the same units as those of the evolutionary distances used to infer the phylogenetic tree. The evolutionary distances were computed using the Maximum composite likelihood method and are in the units of the number of base substitutions per site. All the fungal endophytes recovered in the present study belonged to phylum Ascomycota besides *Rhizopus oryzae* (4.4%: Mucormycotina) that belongs to phylum Zygomycota. Within Ascomycota, endophytic fungal isolates obtained are in six families of class Sordariomycetes (40%: Hypocreaceae, Glomerellaceae, Xylariaceae, Nectriaceae, Diaporthaceae and Chaetomiaceae), two families of class Dothideomycetes (33.3%: Pleosporaceae, Botryosphaeriaceae), two families of class Saccharomycetes (4.4%: Debaryomycetaceae, Trichomonascaceae) and single family of class Eurotiomycetes (16.6%:Trichocomaceae) (Fig 1). *Aspergillus niger* was observed as the most dominant species with 10% relative abundance (RA), followed by *D*. *phaseolorum*, *Hypocrea* sp. and *M*. *phaseolina* (RA = 7.86%) (Table 2). Only 3 endophytic fungal isolates namely, *Bipolaris maydis* with relative abundance (RA) of 2.22%, *Meyerozyma guilliermondii* (RA = 2.2%) and *Fusarium verticillioides* (RA = 1.11%) were exclusively isolated from stem tissues and rest were present in both the tissues (S1 Table and Table 2)

### Spatial and temporal variations in endophytic fungal isolates

**Fig 2:**
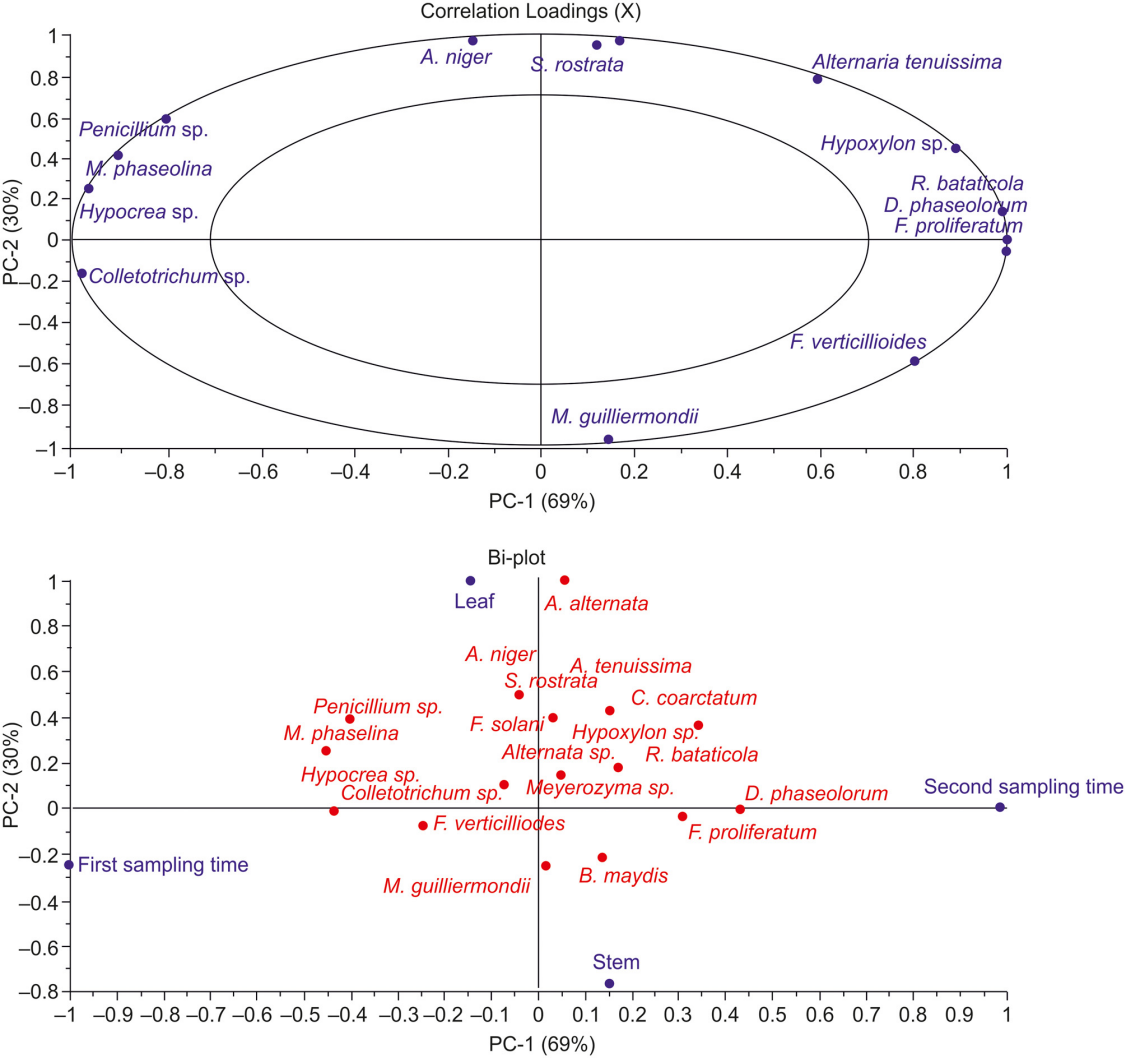
Principal component analysis (PCA) of fungal endophytes isolated from different tissues in distinct sampling time from *O*.*sanctum* performed using Unsrcambler X (version 10, CAMO, USA).

**Fig 3:**
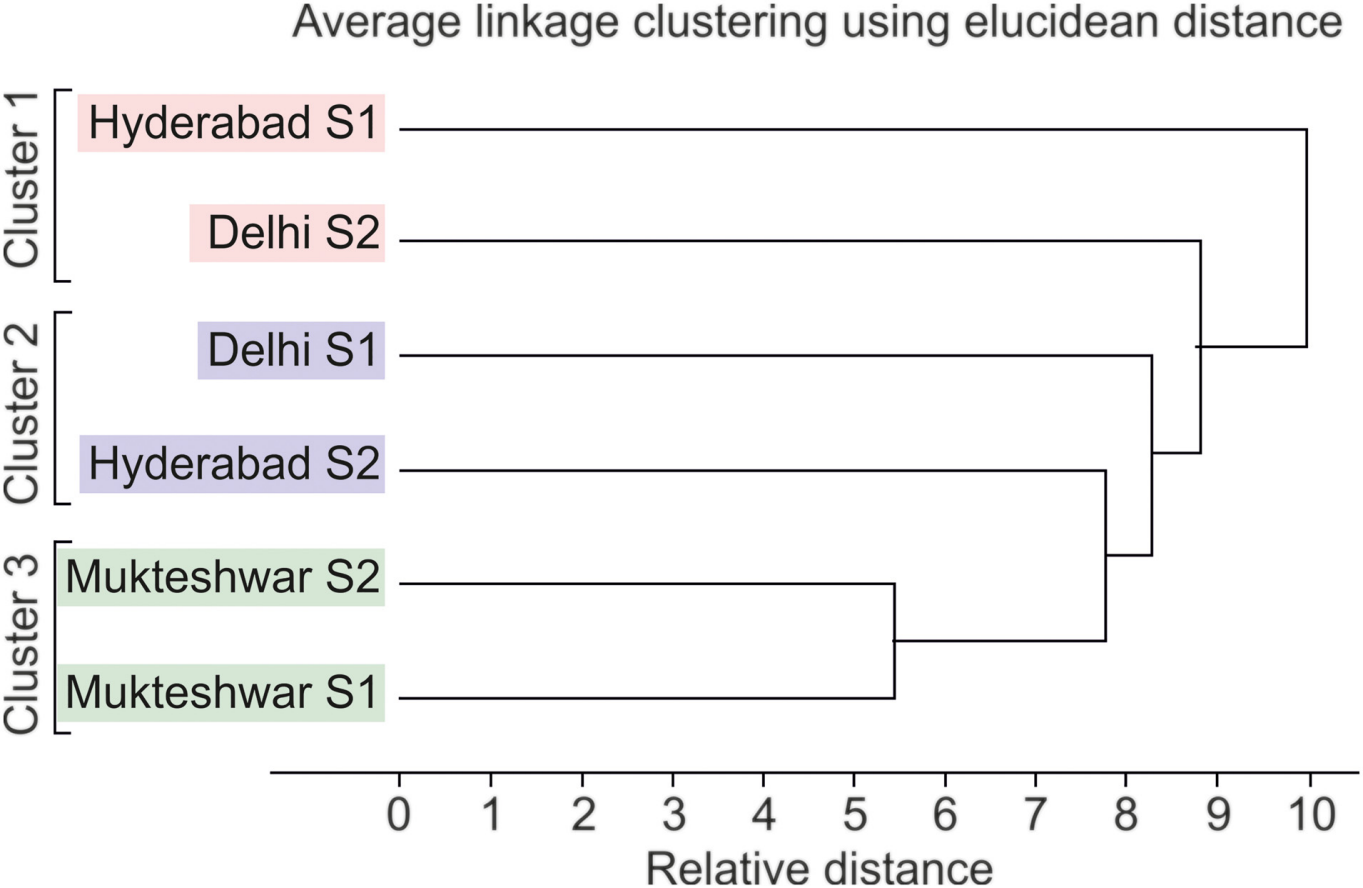
Dendrogram showing variability of endophytic fungal communities based on different geographic locations and mean temperature at the time of sampling produced using Unsrcambler X (version 10, CAMO, USA).

Since medicinal value of *O*.*sanctum* resides largely inside leaf tissues, we incubated more leaf tissues for endophytic fungal isolation. A total of 313 plant tissue segments (222 leaf and 91 stem tissues) of *O*.*sanctum* collected from all geographical locations in both sampling times for isolation of endophytic fungi were incubated (Table 3). We characteristically observed both spatial and temporal variations in endophytic mycobiota.). In leaf, highest isolation rate of fungal endophytes in *O*. *sanctum* (40%) was observed in samples collected from Hyderabad during first sampling time and from Delhi in second collection in 2011. While, in stem tissues Mukteshwar samples collected during first sampling had highest isolation rate (71.4%) followed by isolation rate (50%) from Hyderabad during first collection. *O*. *sanctum* collected in second sampling from Mukteshwar recorded lowest isolation rate of fungal endophytes both for leaf (10.7%) and stem (10%) tissues (Table 3). Bi-plot and correlation loading plot generated by principal component analysis showed that fungal endophytes such as *Alternaria tenuissima*, *A*.*alternaria*, *Macrophomina phaseolina* and *Penicillium* sp. were more abundant in leaf tissues. While *Fusarium verticillioides*, *Bipolaris maydis*, *Chaetomium coarctatum*, *Rhizoctonia bataticola*, *Hypoxylon sp*., *Diaporthe phaseolorum*, *Alternaria tenuissima* were exclusively recovered from second sampling in 2011 suggesting strong seasonal implications on endophytic communities (Fig 2). In addition, when species richness recovered from both plant parts from each site in different sampling time were considered alongwith temperature at the time of sampling for cluster analysis, the resulting Dendrogram revealed concurrence between species richness and mean temperature of the geographical location at the time of plant sampling (Fig 3).

**Table 3:** Isolation rate of endophytic fungi isolated from *Ocimum sanctum*.

	First sampling in 2010 (August-september, 2010)						Second sampling in 2011 (April-June, 2011)					
	Delhi	Delhi	Hyderabad	Hyderabad	Mukteshwar	Mukteshwar	Delhi	Delhi	Hyderabad	Hyderabad	Mukteshwar	Mukteshwar
	Leaf	Stem	Leaf	Stem	Leaf	Stem	Leaf	Stem	Leaf	Stem	Leaf	Stem
**Incubated plant segments**	36	15	30	8	35	7	35	21	28	20	28	20
**Endophytic fungal isolates**	10	5	12	4	8	5	14	4	8	15	3	2
**Isolation Rate (%)**	22.7	33.3	40	50	22.8	71.4	40	19.1	28.6	75	10.7	10

### Diversity, richness and similarity index

Shannon diversity in the first collection is highest in Hyderabad leaf tissues (H' = 1.907) whereas in second collection it was highest from leaf tissues of Delhi (H' = 1.846). Shannon diversity has been recorded to be higher in leaf tissues as compared to stem tissues at all places besides Hyderabad in second sampling time (Table 4). The species richness determined by calculating the Menhinick’s index (Dmn) revealed that the leaves collected from Hyderabad in first sampling were richest in endophytic fungal species (Dmn 3.46), followed by the leaves from Delhi in second sampling in 2011 (Dmn 1.87), and finally amongst stem tissues Hyderabad in second sampling in 2011 (Dmn 1.656) had a rich endophytic assemblage. Camargo’s index depicting the tissue-specific fungal dominance was 0.746 for the Delhi stem tissues in first sampling (highest), followed by that of leaves of Hyderabad in second sampling 0.709 (Table 4). Combined data obtained by these mathematical tests indicate that diversity of fungal endophytes harbored inside leaf tissues was higher than in stem tissues of *O*. *sanctum*. Pielou's eveness index, measuring the variation in endophytic mycopopulation differs among geographic locations ranged between 0.456 to 1. Pielou's eveness index differs correspondingly amongst geographical locations but is nearly similar when compared between plant tissues solitary (Table 4). Species shared between Delhi and Hyderabad was highest in second sampling which resulted in high Sorenson’s similarity index (0.33). Collectively analysed, lower similarity index amongst three sites indicates uniqueness of endophytic mycobiota in different sampling times from different locations, observations which are further substantiated by PCA (Table 5).

**Table 4:** Diversity indices of endophytic fungi isolated from *Ocimum sanctum*.

	First sampling in 2010 (August-september, 2010)						Second sampling in 2011 (April-June, 2011)					
	Delhi	Delhi	Hyderabad	Hyderabad	Mukteshwar	Mukteshwar	Delhi	Delhi	Hyderabad	Hyderabad	Mukteshwar	Mukteshwar
	Leaf	Stem	Leaf	Stem	Leaf	Stem	Leaf	Stem	Leaf	Stem	Leaf	Stem
**Shannon diversity (H')**	1.0519	0.950	1.907	0.5623	1.213	1.039	1.846	1.039	1.328	1.656	-	0.693
**Menhinick's species richness (Dmn)**	1.58	1.34	3.46	1	1.515	1.6	1.87	1.5	1.41	1.656	-	1.41
**Pielou's eveness index (J')**	0.4568	0.590	0.768	0.406	0.5833	0.75	0.699	0.75	0.6352	0.611	-	1
**Camargo's index (1/Dmn)**	0.632	0.746	0.289	1	0.660	0.625	0.534	0.666	0.7092	0.645	-	0.709

**Table 5:** Comparison of different similarity indices among different collection sites and time.

	First sampling in 2010 (August-september, 2010)			Second sampling in 2011 (April-June, 2011)		
	Species Shared	Jaccard’s SI	Sorensen’s SI	Species Shared	Jaccard’s SI	Sorensen’s SI
S1 vs S2	2	0.194	0.324	3	0.463	0.633
S2 vs S3	2	0.25	0.311	None	0	0
S3 vs S1	1	0.185	0.3125	None	0	0

S1-Delhi; S2-Hyderabad; S3-Mukteshwar.

### Antiphytopathogenic activity

**Fig 4:**
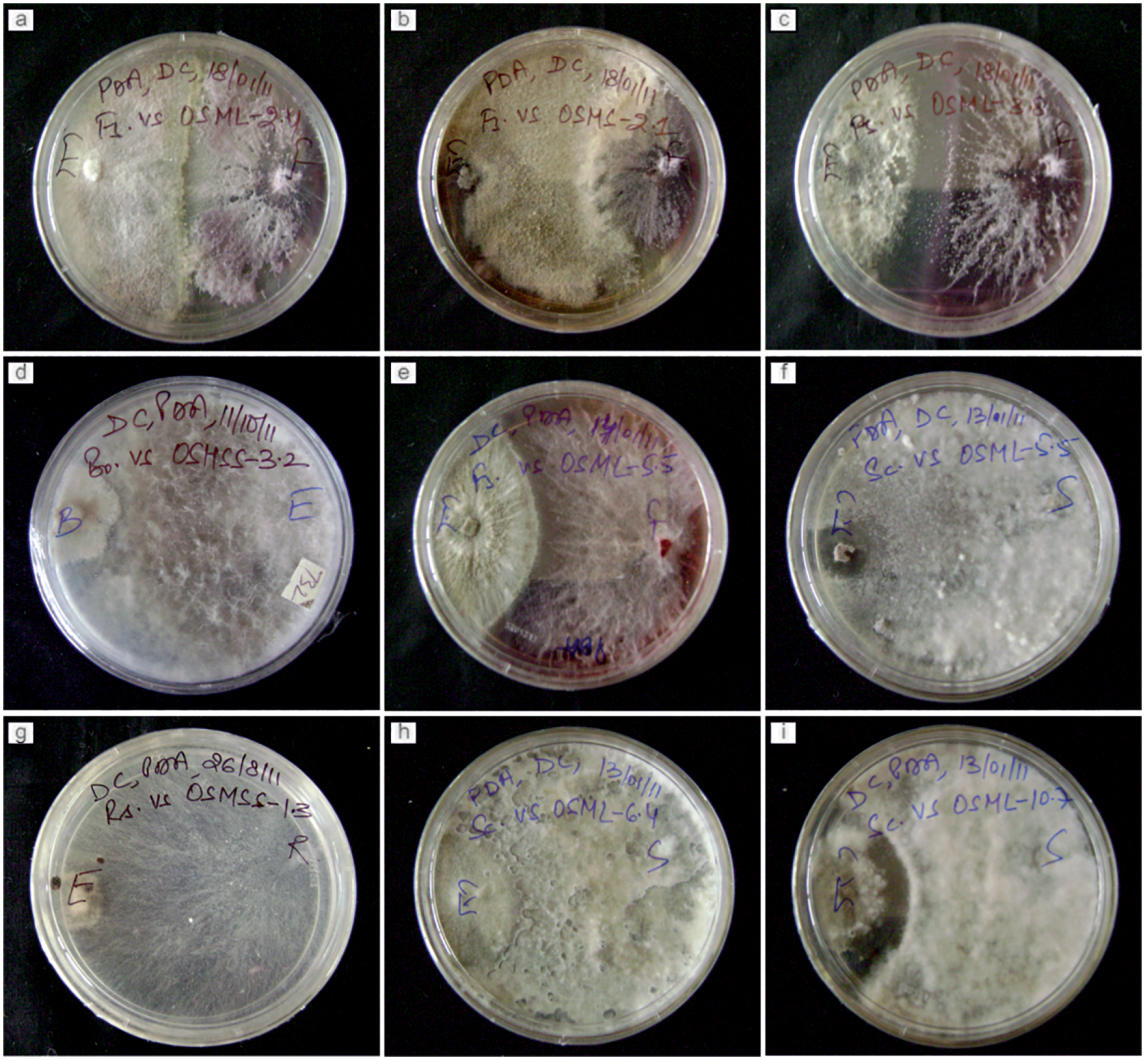
Types of endophyte-host pathogen interactions observed in dual culture/confrontation assay.

Vital observations were made to gain insight into the biocontrol potential of endophytic fungi outside its plant source on an artificial media *in vitro* against four widespread phytopathogens in India namely *Botrytis cinerea*, *Sclerotinia sclerotiorum*, *Fusarium oxysporum* and *Rhizoctonia solani*. After doing necessary macroscopic evaluation of the interaction, we followed literature sources available on endophyte-pathogen interaction types and categorised dual culture bioassay results accordingly [43], [39]. Mostly results obtained were similar within the replication plates between all endophytic fungi against all four pathogenic fungi, but in certain cases of conflict, we accepted the observations as appeared in at least 2 out of 3 replication plates. Based on our results we categorized mode of interactions into nine (I-IX) types. The key purpose of the dual culture bioassay was based on bioprospecting strategy to select potential endophytes with having antiphytopathogenic activity. The means of achieving the objective was to carry out qualitative screening of plethora of fungal endophytes recovered from different medicinal plant hosts in distinct sampling times on the basis of their ability to exhibit antagonism against broad spectrum phytopathogens. The selected fungal endophytes were marked and maintained for further evaluation (Table 6, Fig 4). Nearly 23% of the total fungal isolates were considered as potent biocontrol agent, as they showed antifungal activity against at least three of the broad spectrum plant pathogens (S2 Table).

**Table 6:** Different modes of interaction observed between isolated endophytic fungi and the four plant pathogens in dual culture/Confrontation bioassay.

**Class I**	Both pathogen and endophyte grow towards each other, growth stopped at equidistant at physical contact but mycelial boundary is maintained by both even after two weeks of observation
**Class II**	Both pathogen and endophyte grow towards each other, but endophyte grows over the pathogenic fungi mycelia; pushing it backwards
**Class III**	Both pathogen and endophyte grow towards each other, zone of inhibition is formed which is maintained for another week
**Class IV**	Pathogenic fungi’s growth is completely stunted by endophytic fungi at its periphery
**Class V**	Both pathogen and endophyte grow towards each other and Pathogenic fungi overgrows endophytic and stops its growth at physical contact
**Class VI**	Pathogenic fungi completely stunts the growth of endophytic fungi at its periphery
**Class VII**	Both pathogen and endophyte grow towards each other, but pathogenic fungi mycelia overgrows endophytic fungi’s mycelia
**Class VIII**	Both pathogen and endophyte grow towards each other, but pathogenic fungi grow over endophytic fungi at the physical contact zone only
**Class IX**	Both pathogen and endophyte grow; but circumference covered by pathogenic fungi is much more than endophytic fungi and zone of inhibition is formed, pathogenic fungi secretes metabolites inhibiting further mycelial growth of endophytic fungi

### Bioactivity of crude extracts of fungal endophytes

Based on the results of dual culture bioassay (S2 Table), following thirteen: Ocl-13.1’(*Penicillium* sp.), OSHL-5.2 (*Alternaria* sp.), OSHL-2.1 (*M*. *phaseolina*), OSHS-3.1(*M*. *phaseolina*), OSML-5.4 (*Colletotrichum* sp.), OSMS-2.2 (*Aspergillus niger*), OSMS-2.3 (*Meyerozyma guilliermondii*), OSDSL-9.8 (*Fusarium proliferatum*), OSDSS-2.5 (*Chaetomium coarctatum*), OSDSL-5.6 (*Alternaria alternata*), OSHSS-5.2 (*Sympodiomyces* sp.), OSHSS-1.3 (*Fusarium proliferatum*) OSHSS-2.3 (*Diaporthe phaseolorum*) endophytic fungal isolates were multiplied on rice medium, and their crude ethylacetate extracts were subjected to intoxicated/poisoned food bioassay to investigate antiphytopathogenic activity of crude fungal extracts against all four phytopathogens. IC_50_ values of ethylacetate crude extracts of selected thirteen endophytic fungal isolates at five different concentrations was calculated by regression analysis are summarised as Table 7. Extract of *Chaetomium coarctatum* recorded the highest activity against all phytopathogens with IC_50_ value of < 1mg/ml ranging from 0.262mg/ml– 0.553 mg/ml. Second best activity was reported with *Fusarium proliferatum* obtained from Hyderabad with IC_50_ value ranging from 0.299 to 1.04 mg/ml.

**Table 7:** IC_50_ value (mg/ml/) of crude ethylacetate extracts of endophytic fungi recovered from *O*. *sanctum* calculated by Regression equation analysis.

Endophytic fungi	Location/sampling year/source	*B*. *cinerea*	*S*. *sclerotiorum*	*F*. *oxysporum*	*R*. *solani*
*Penicillium* sp. (Ocl-13.1’)	Delhi/2010/leaf	5.536	4.68	9.624	5.538
*Alternaria* sp. (OSHL-5.2)	Hyderabad/2010/leaf	1.422	0.736	31.84	1.265
*Macrophomina phaseolina* (OSHL-2.1)	Hyderabad/2010 /leaf	4.504	-	1.97	1.41
*Macrophomina phaseolina* (OSHS-3.1)	Hyderabad/2010 /Stem	1.723	2.93	0.599	-
*Colletotrichum* sp.(OSML-5.4)	Mukteshwar/2010/leaf	1.029	5.97	2.62	3.93
*Aspergillus niger* (OSMS-2.2)	Mukteshwar/2010/stem	0.834	1.78	2.021	0.972
*Meyerozyma guilliermondii* (OSMS-2.3)	Mukteshwar/2010/stem	0.225	0.144	1.286	1.47
*Fusarium proliferatum* (OSDSL-9.8)	Delhi/2011/leaf	2.277	4.98	1.611	5.161
*Chaetomium coarctatum* (OSDSS-2.5)	Delhi/2011/stem	0.456	0.369	0.553	0.262
*Alternaria alternata* (OSDSL-5.6)	Delhi/2011/leaf	0.965	2.090	0.076	3.045
*Fusarium proliferatum* (OSHSS-1.3)	Hyderabad/2011/leaf	0.299	-	0.509	1.04
*Diaporthe phaseolorum* (OSHSS-2.3)	Hyderabad/2011/stem	1.957	5.22	2.216	3.632
*Sympodiomyces s*p.(OSHSS-5.2)	Hyderabad/2011/stem	4.90	0.564	6.12	1.15

Strain specificity for bioactivity was strongly observed. For example, *Fusarium proliferatum* obtained from Hyderabad exhibited IC_50_ value of 0.51mg/ml against *F*.*oxysporum* which was stronger than another isolate of *F*. *proliferatum* recovered from Delhi in second sampling (Table 7). Likewise, *Macrophomina phaseolina* recovered from stem tissue displayed IC_50_ value of 1.723mg/ml against *B*. *cinerea* whereas *Macrophomina phaseolina* isolated from leaf tissue exhibited IC_50_ value of 4.504 mg/ml against *B*.*cinerea*. Hexane extract of *M*. *phaseolina* recovered from leaf tissue demonstrated highest activity against *S*. *sclerotiorum* with IC_50_ value of 0.38 mg/ml.

### Metabolite screening by GC-MS

**Fig 5:**
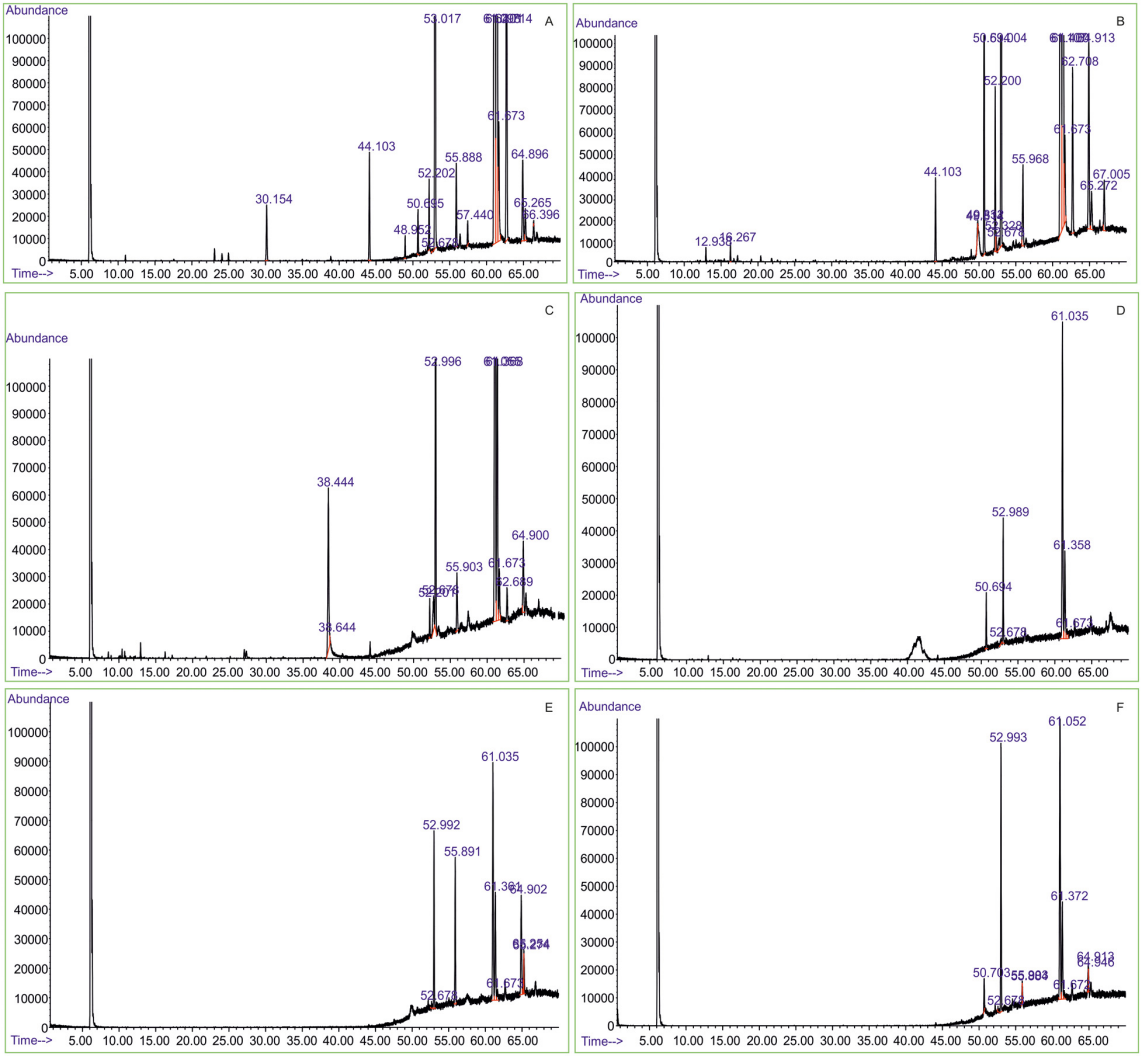
A: GC-Chromatogram of *Macrophomina phaseolina* recovered from leaf tissue (OSHL-2.1)- major peaks: 30.156 (2H-Pyran-2-one,5,6-dihydro-6-pentyl), 53.017(Hexadecanoic acid), 64.986 (Linoleic acid), 65.265(10-Octadeconic acid)B: *Macrophomina phaseolina* recovered from stem tissue-(OSHS-1.3) major peaks: 61.108 (9,12-Octadecadieonic acid, methyl ester), 55.970(Trifluoroacetoxy hexadecane). C:-*Chaetomium coarctatum* (OSDSS-2.5)- major peaks: 38.644 (2-Fluorobenzoic acid), 38.444 (2,5-Difluorobenzoic acid), 64.900 (Linoleic acid, ethyl ester), D: *Alternaria* sp (OSHL-5.2).-major peaks: 52.990(Hexadecanoic acid), 61.036 (9,15- Octadecadienoic acid, methyl ester) E-*Fusarium proliferatum*(OSHSS-1.3) major peaks: 52.992(Hexadecanoic acid), 64.902(Linoleic acid), 65.954(Oleic acid); F-*Colletotrichum* sp.-(OSML-5.4) major peaks: 61.372(7-Hydroxy3-(1,1-dimethylprop-2-enyl) coumarin), 61.052(9,12-Octadecadieonic acid).

Fungal crude extracts of OSHL-2.1(*M*. *phaseolina*), OSHS-3.1 (*M*.*phaseolina*), OSDSS-2.5 (*Chaetomium coarctatum*), OSHL-5.2 (*Alternaria* sp.), OSML-5.4 (*Colletotrichum* sp.) and OSHSS-1.3 (*F*. *proliferatum*) were found positive for terpenoids in preliminary phytochemical screening. GC-MS chromatography of hexane extracts of these selected endophytic fungal isolates revealed presence of volatile compounds, fatty acids, and aliphatic constituents. GC-MS chromatogram of *M*. *phaseolina* (OSHL-2.1) showed presence of 2H-Pyran-2-one, 5, 6-dihydro-6-pentyl at RT 30.156, hexadecanoic acid at RT 53.017, linoleic acid at RT 64.986 (Fig 5A) while hexane extract of another strain of *M*. *phaseolina* (OSHS-3.1) showed presence 9,12-octadecadieonic acid, methyl ester at RT 61.108, trifluoroacetoxy hexadecane at RT 55.970 as major peaks reflecting strain variability (Fig 5B). In GC-MS chromatogram of hexane extract of *Chaetomium coarctatum* (OSDSS-2.5) 2-fluorobenzoic acid at RT 38.644, 2,5-difluorobenzoic acid at RT 38.444, linoleic acid, ethyl ester at RT 64.900 were observed as major peaks (Fig 5C). In *Alternaria* sp. (OSHL-5.2) hexane extract, hexadecanoic acid at RT 52.990, 9,15- octadecadienoic acid, methyl ester at RT 61.036 were recorded as major peaks (Fig 5D) while that of *Fusarium proliferatum* (OSHSS-1.3)-major peaks were hexadecanoic acid at RT 52.992, linoleic acid at RT 64.902, oleic acid at RT 65.954 (Fig 5E). *Colletotrichum* sp. (OSML-5.4) major peaks recorded were 7-hydroxy3-(1, 1-dimethylprop-2-enyl) coumarin at RT 61.372, 9, 12-octadecadieonic acid at RT 61.052 (Fig 5F).

### Bioassay of pure compounds

**Fig 6:**
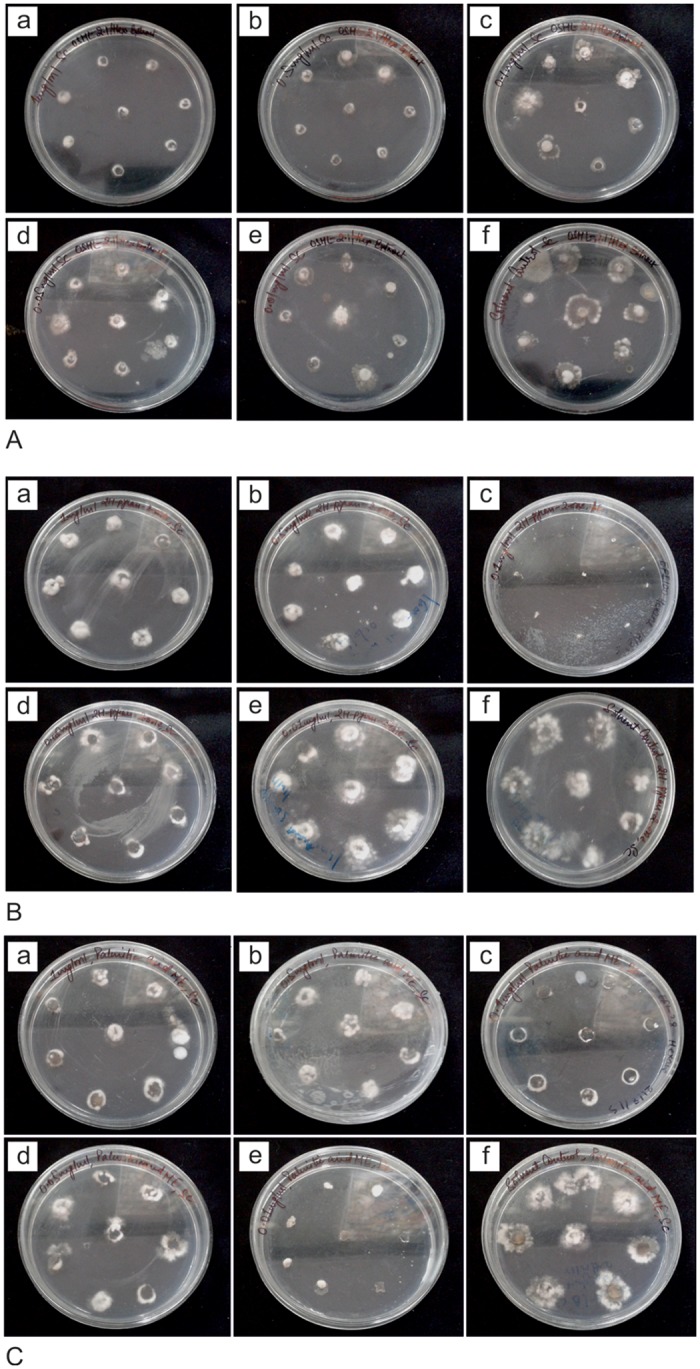
Poisoned food bioassay at five different concentrations [1mg/ml (a), 0.5mg/ml (b), 0.1mg/ml (c), 0.05mg/ml (d), 0.01mg/ml (e) of treatment and solvent control (f)] of A. *M*. *phaseolina* (OSHL-2.1) hexane extract against *S*. *sclerotiorum* B. 5,6-Dihydro 2H-pyran-2-one-6 pentyl against *S*. *sclerotiorum* C. palmitic acid methyl ester against *S*. *sclerotiorum*.

Among all the compounds recorded in GC-MS Chromatography of hexane extracts of fungal endophytes 2H-pyran-2-one, 5,6-dihydro-6-pentyl, palmitic acid, methyl ester, oleic acid are reported to have antifungal activity [44], [45]. Therefore, in order to confirm the contributory effect of these compounds on the antifungal activity of hexane extracts of endophytic fungi, they were procured from commercial sources and their antifungal activity was evaluated against phytopathogens. 2H-pyran-2-one, 5, 6-dihydro-6-pentyl and palmitic acid, methyl ester exhibited antiphytopathogenic activity exhibited IC_50_ value of 1.002 and 0.662 respectively against *S*. *sclerotiorum* confirming their antifungal activity (Table 8, Fig 6).

**Table 8:** IC_50_ value (mg/ml/) of crude hexane extract of *M*. *phaseolina* (OSHL-2.1) and pure compounds at five different concentrations calculated by Regression equation analysis.

Endophytic fungi/ Pure Compound	*B*. *cinerea*	*S*. *sclerotiorum*	*F*. *oxysporum*	*R*. *solani*
*M*. *phaseolina* (OSHL-2.1)	0.994	0.38	0.867	0.884
2H-pyran-2-one, 5,6-dihydro-6-pentyl	3.006	1.002	0.27	1.147
Oleic acid methyl ester	2.079	0.547	0.27	1.228
Palmitic acid methyl ester	2.332	0.662	0.264	1.729

## Discussion

All the fungal endophytes reported from *O*. *sanctum* in present study belong to Ascomycota besides *Rhizopus oryzae* (Zygomycota). *Rhizopus oryzae* has been reportedly primary causal agent of tuber rot disease in sweetpotato in India and in this study has been isolated from Delhi and Hyderabad in first sampling season [46]. Ascomycota is the largest phylum of fungi, one of the most diverse and ubiquitous phyla of eukaryotes covering approximately 8% of the Earth’s landmasses [47]. Interestingly, all the fungal endophytes recovered from *O*. *sanctum* have been known as plant pathogens. The study for the first time reports following three plant pathogens as endophytes, *Bipolaris maydis*, *Rhizoctonia bataticola* and *Chaetomium coarctatum* harbored inside *O*. *sanctum*. Earlier literature suggests that endophytes have evolved directly from plant pathogenic fungi [48], [49]. It has been established for a plethora of fungi that pathogenic-endophytic lifestyles are interchangeable and are due to a number of environmental, chemical and/or molecular triggers [50], [51], [52]. Thus, even a fungus that is pathogenic in one ecological niche can be endophytic to plant hosts in another ecosystem. Photita et al in 2004 confirmed that fungal endophytes isolated from wild banana (*Musa acuminata*) were *in vitro* latent pathogens [53]. Fungal endophytes reported from present study have been previously described in their endophytic nature producing bioactive secondary metabolites. For instance, *Fusarium proliferatum* causing root rot on Soybean (*Glycine max*) [54], has been reported to produce a plethora of secondary metabolites with different biological activities. It has been known to produce sanguinarine possessing antibacterial, anthelmintic, and anti-inflammatory properties when isolated from *Macleaya cordata* [55]. 240ng/l of paclitaxel was produced by*Fusarium proliferatum* when isolated from paclitaxel producing plant *Taxus x media* [56]. Two new tricyclic sesterterpenes, fusaprolifins A and B were reportedly produced from the same fungi when isolated from marine mangrove plant *Bruguiera sexangula* [57]. *Alternaria alternata* is a well known necrotrophic fungus [58]. Altenuene derivatives, isocoumarin alongwith other metabolites were produced by *Alternaria alternata* isolated from *Camellia sinensis* [59]. Diketopiperazines extracted from grapevine endophyte *Alternaria alternata* effectively inhibited *Plasmopara viticola* [60]. Also, one of the endophytic strain of *Alternaria alternata* harbored inside fruit and seeds of *Miquelia dentate* Bedd. produced camptothecin, 9-methoxy camptothecin and 10-hydroxy camptothecin [61]. Similarily, *Penicillium crustosum* recently described as causal agent of blue mold in stored apples [62]. In its endophytic state, when recovered from seeds of coffee beans it reportedly produced mycophenolic acid and a new phthalide, 5-hydroxy-7-methoxy-4-methylphthalide [63].

In the present study Mukteshwar (altitude: 7500 feet) recorded least isolation rates in second sampling time which is in line with the report of Carroll and Carroll [64] who reported low isolation rates at high elevations that could result from delayed onset of endophytic infections. Highest isolation frequency (75%) was recovered from stem tissues from Hyderabad collected in second sampling season. One possible explanation could be that recently penetrated epiphytic hyphae could have survived surface sterilization procedures [65]. Bertoni and Cabral [66] found that epiphytic fungi may form limited colonizations through substomatal chambers and also avoid the effects of surface sterilization. In this study, only the culturable endophytic fungi could be isolated and the assemblages do not represent non-culturable endophytic fungal isolates of the *O*.*sanctum* plants investigated. It should also be mentioned here that rDNA ITS analysis can sometimes underestimate the endophytic fungal ‘species diversity’, and additional parameters should be coupled to ITS rDNA sequence [67]. Increased abundance of *A*. *tenuissima*, *A*.*alternaria*, *M*. *phaseolina* and *Penicillium* sp. in leaf tissues could be described as tissue preference. Tissue preference has been previously reported for endophytic fungi, highlighting their surviving ability within a specific substrate [34]. Occurrence of eight phytopathogens viz. *F*. *verticillioides*, *B*. *maydis*, *C*. *coarctatum*, *R*. *bataticola*, *Hypoxylon sp*., *D*. *phaseolorum* and *A*. *tenuissima* together in second sampling as explained in principle component analysis, could be hypothesised that may be during high temperature and low humidity plant pathogens are less virulent and are better established as endophyte within plant tissues coping with medicinal plant’s defence mechanism [68].

Endophytic fungal diversity is higher in tropical and subtropical plants than other climatic zones likewise reported from Delhi and Hyderabad regions in the present study [69], [70], [71], and [72].

Additionally, cluster analysis inferred distribution and abundance of endophytic fungal species from different geographical locations as a function of temperature. It grouped together species richness of Delhi (second sampling) and Hyderabad (first sampling) when temperature ranged from 23–24°C into cluster 1, cluster 2 comprises of Delhi (first sampling) and Hyderabad (second sampling) when temperature ranged from 30–31°C and cluster 3 belongs to endophytic mycobiota of Mukteshwar where temperature range was 14–17°C during both sampling times. This revelation is in accordance with previous studies elucidating higher influence of temperature, precipitation and other climatic factors than geography on endophytic communities of plants [73], [74].

Following on the lines of “bioprospecting” and “biological diversity can lead to novel chemistry” from endophytic fungi of medicinal plants [1], [9], [24] all the endophytic fungal isolates recovered were tested for antiphytopathogenic activity by means of dual culture/confrontation bioassay against four economically significant plant pathogens. *S*. *Sclerotiorum* causes heavy yield losses up to the tune of 40% in Brassicas in India [75]. *Fusarium* wilt is one of the major diseases of chickpea and at national level the yield losses encountered was reported to the tune of 60 per cent in India [76]. Botrytis grey mould (BGM), caused by the fungus *Botrytis cinerea* Pers. ex Fr., is an important disease of chickpea causing economic losses across the world in chickpea-growing regions. There are no available resistance sources in cultivated chickpea against this disease [77]. *R*. *solani* causes economically important root and hypocotyl diseases in common bean throughout the world [78]. Different degrees and types of interactions were observed between phytopathogens and endophytic fungal isolates. Each one of the 90 endophytic fungal isolate of *O*. *sanctum* exhibited antagonism against atleast one plant pathogen. For instance, all the endophytic fungal isolates obtained from Delhi in first sampling in 2010 showed antiphytopathogenic activity against *Fusarium oxysporum*. Best antiphytopathogenic activity was depicted from endophytic fungal isolates of Hyderabad and Mukteshwar in first sampling in 2010. 50% of the endophytic fungal isolates were found to be active against *Rhizoctonia solani* majority of them identified as *Diaporthe phaseolorum* collected from Hyderabad in second sampling in 2011. In addition, *Penicillium* sp.isolated from Mukteshwar in first sampling in 2010 demonstrated antiphytopathogenic activity against all of plant pathogens tested whereas other isolate identified *Penicillium* sp. recovered from Hyderabad in first sampling in 2010 could only be antagonistic against *S*. *sclerotiorum*. Thus, our report corroborates that biology of endophytes may be influenced by many factors such as localization, season and environment [79].

Crude ethylacetate extracts of *M*. *phaseolina* (OSHL-2.1) exhibited effective inhibitory activity against *B*. *cinerea*, *F*. *oxysporum* and *R*. *solani* with 4.504 mg/ml, 1.97mg/ml and 1.41mg/ml IC_50_ values respectively. Whereas hexane extract of *M*. *phaseolina* (OSHL-2.1) demonstrated highest activity against *S*. *sclerotiorum* with IC_50_ value of 0.38 mg/ml followed by *F*. *oxysporum* with IC_50_ value of 0.867mg/ml. Preliminary identification of bioactive metabolites were conducted on crude hexane extracts.GC-MS chromatogram of hexane extract of *M*. *phaseolina* illustrated presence of 2H-pyran-2-one, 5, 6-dihydro-6-pentyl at RT 30.152 and palmitic acid, methyl ester at RT 53.017, while that of *C*. *coarctatum* (OSDSL-2.5) illustrated Oleic acid at RT 61.675. Present study for the time reports antifungal activity of extracts of *M*. *phaseolina* and *C*. *coarctatum*. Antifungal activities of above mentioned pure compounds against plant pathogens resulted in remarkable revelation. Oleic acid methyl ester, palmitic acid methyl ester and 5, 6 Dihydro-2H-pyran-2-one-6 pentyl exhibited strong inhibitory activity against *S*. *sclerotiorum* with 0.547mg/ml, 0.662mg/ml and 1.002 mg/ml IC_50_ value while against *F*. *oxysporum* it was 0.27 mg/ml, 0.264 mg/ml and 0.27mg/ml respectively. Thereby, buttressing the fact that 2H-pyran-2-one, 5,6-dihydro-6-pentyl and palmitic acid, methyl ester are bioactive metabolites synthesised by endophytic fungus *M*. *phaseolina* which are responsible for its antiphytopathogenic activity against *S*. *sclerotiorum*. Antifungal activity of 6-Pentyl-2H-pyran-2-one and its analogs has been well documented in literature [44], [80]. Reportedly, 6-Pentyl-2H-pyran-2-one displayed highest antifungal activity against *Penicillium* spp [81]. Another study reported its production from *Trichoderma harzianum* strains isolated from Himalayan region exhibiting antifungal activity phytopathogenic fungi [82]. The production of 2H-pyran-2-one, 5,6-dihydro-6-pentyl from *M*. *phaseolina* an endophytic fungus is being reported for the first time. Likewise, GC-MS chromatogram of hexane extracts revealed presence of aliphatic constituents such as 9-hexadecenoic acid, octadeconoic acid, oleic acid, linoleic acid among others. These aliphatic constituents have been widely described as antifungal in various studies [83]. Hexadecanoic acid and 9, 12 octadecadienoic acid identified from extracts of *T*. *crispa* were found to be strongly active against *C*. *albicans* [84]. The major bioactive compound oleic acid found in *L*. *cristata* was reported be highly effective to phytopathogens *Colletotrichum fulcatum* NCBT 146, *Fusarium oxysporum* NCBT 156 and *Rhizoctonia solani* NCBT 196 [85]. Another study described inhibitory activity of linolenic and linoleic acids against phytopathogenic fungi including *R*. *solani* [45].

## Conclusion

The study reports noted plant pathogens such as *Bipolaris maydis*, *Rhizoctonia bataticola* and *Chaetomium coarctatum* in endophytic state harbored inside *O*. *sanctum* for the first time. In present study metabolites produced by *M*. *phaseolina* recovered from Hyderabad in first collection has exhibited promising antifungal activity against *S*. *sclerotiorum* and *F*. *oxysporum* broad spectrum phytopathogens. Metabolites originated from fungal endophytes hold promise to be further developed as greener and safer biocontrol agent in crop disease management. It can be concluded that fungal endophytes harbored inside leaf and stem tissues of *Ocimum sanctum* collected from different geographical locations in different sampling times hold great promise not only as biocontrol agents against broad spectrum and economically significant phytopathogens, but also as sustainable resource of novel antifungal secondary metabolites.

## Supporting Information

S1 Fig Cash bill from Herbal Garden at ANGR agricultural University, Hyderabad. (TIFF)

S1 Table Number of endophytic fungal isolates harbored in leaf and stem tissues of *Ocimum sanctum*. (DOCX)

S2 Table Dual culture bioassay results of Endophytic fungi isolated from *O*. *sanctum* against phytopathogens. (DOCX)
