# Supplementary figures and images for: Fungal Endophyte Diversity and Bioactivity in the Indian Medicinal Plant Ocimum sanctum Linn
Source: PLoS One. 2015 Nov 3;10(11):e0141444. doi: 10.1371/journal.pone.0141444 (PMC4631451; doi:10.1371/journal.pone.0141444)

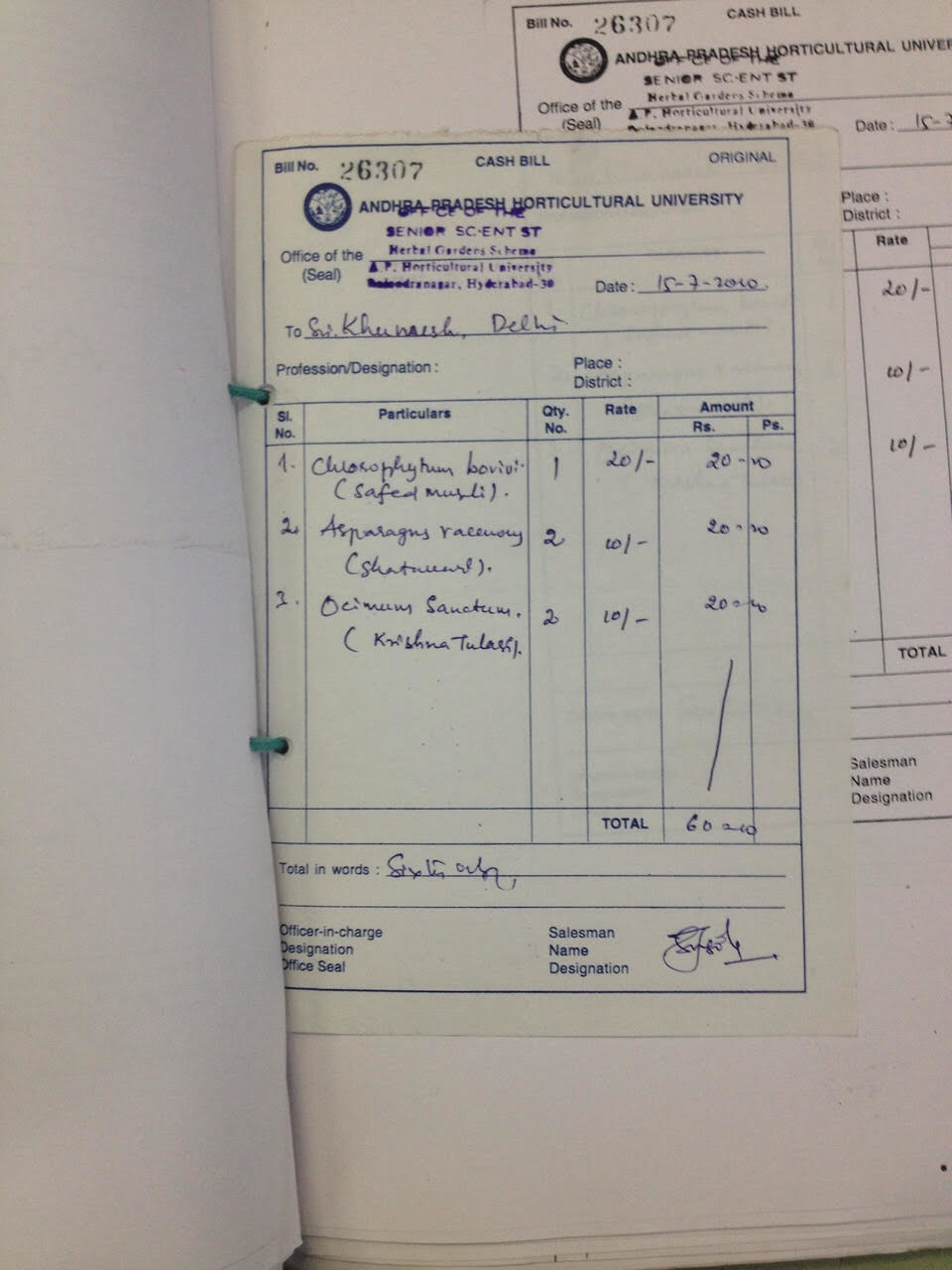

Supplement: S1 Fig — (TIFF) [file pone.0141444.s001.tiff]
