# Supplementary material for: Fungal Endophyte Diversity and Bioactivity in the Indian Medicinal Plant Ocimum sanctum Linn
Source: PLoS One. 2015 Nov 3;10(11):e0141444. doi: 10.1371/journal.pone.0141444 (PMC4631451; doi:10.1371/journal.pone.0141444)
Supplement: S1 Table — (DOCX) [file pone.0141444.s002.docx]

**Fungal endophyte diversity and bioactivity in the Indian medicinal plant *Ocimum sanctum*** **Linn**.

**Kanika Chowdhary**1 and **Nutan Kaushik**2*

1TERI University, 10th Institutional Area, Vasant Kunj, New Delhi-110070, India

2The Energy and Resources Institute (TERI), India Habitat Center, Lodhi Road, New Delhi – 110003, India

* Corresponding author: Nutan Kaushik, TERI, IHC, Lodhi Road, New Delhi – 110003, India

Phone: 91-11-24682100/ 24682111, Fax: 91-11-24682144/24682145, Email: kaushikn@ teri.res.in

**S1 Table** Number of endophytic fungal isolates harbored in leaf and stem tissues of *Ocimum sanctum*

|  | **First sampling in 2010 (August-september, 2010)** | | | | | | **Second sampling in 2011 (April-June, 2011)** | | | | | |
| --- | --- | --- | --- | --- | --- | --- | --- | --- | --- | --- | --- | --- |
|  | **Delhi** | **Delhi** | **Hyderabad** | **Hyderabad** | **Mukteshwar** | **Mukteshwar** | **Delhi** | **Delhi** | **Hyderabad** | **Hyderabad** | **Mukteshwar** | **Mukteshwar** |
| **Endophytic Fungi** | **Leaf** | **Stem** | **Leaf** | **Stem** | **Leaf** | **Stem** | **Leaf** | **Stem** | **Leaf** | **Stem** | **Leaf** | **Stem** |
| *Alternaria alternata* | 3 |  |  |  |  |  | 2 |  |  |  |  |  |
| *Alternaria sp.* |  |  | 1 |  |  |  | 1 | 1 |  |  |  |  |
| *Alternaria tenuissima* |  |  |  |  |  |  |  |  | 3 |  |  |  |
| *Aspergillus niger* |  |  | 2 |  | 1 | 2 |  |  |  |  | 3 | 1 |
| *Bipolaris maydis* |  |  |  |  |  |  |  |  |  | 2 |  |  |
| *Chaetomium coarctatum* |  |  |  |  |  |  | 4 | 2 |  |  |  |  |
| *Colletotrichum sp.* |  |  |  |  | 2 | 2 |  |  |  |  |  |  |
| *Curvularia lunata* |  |  |  |  | 1 |  |  |  |  |  |  |  |
| *Diaporthe phaseolorum* |  |  |  |  |  |  | 1 |  | 2 | 4 |  |  |
| *Fusarium proliferatum* |  |  | 1 |  |  |  | 1 |  | 1 | 3 |  |  |
| *Fusarium solani* | 1 |  |  |  |  |  |  |  |  |  | 1 |  |
| *Fusarium verticillioides* |  |  |  |  |  |  |  |  |  |  |  | 1 |
| *Hypocrea sp.* | 4 | 3 |  |  |  |  |  |  |  |  |  |  |
| *Hypoxylon sp.* |  |  |  |  |  |  | 2 | 1 |  |  |  |  |
| *Macrophomina phaseolina* |  |  | 4 | 3 |  |  |  |  |  |  |  |  |
| *Meyerozyma guilliermondii* |  |  |  |  |  | 1 |  |  |  | 1 |  |  |
| *Meyerozyma sp.* |  |  | 1 |  |  |  |  |  |  |  |  |  |
| *Penicillium crustosum* | 1 |  |  |  |  |  |  |  |  |  |  |  |
| *Penicillium sp.* |  | 1 |  |  | 4 |  |  |  |  |  |  |  |
| *Rhizoctonia bataticola* |  |  |  |  |  |  | 1 |  | 2 | 3 |  |  |
| *Rhizopus oryzae* | 1 | 1 | 1 | 1 |  |  |  |  |  |  |  |  |
| *Setosphaeria rostrata* |  |  | 1 |  |  |  | 2 |  |  |  |  |  |
