# Supplementary material for: Fungal Endophyte Diversity and Bioactivity in the Indian Medicinal Plant Ocimum sanctum Linn
Source: PLoS One. 2015 Nov 3;10(11):e0141444. doi: 10.1371/journal.pone.0141444 (PMC4631451; doi:10.1371/journal.pone.0141444)
Supplement: S2 Table — (DOCX) [file pone.0141444.s003.docx]

**Fungal endophyte diversity and bioactivity in the Indian medicinal plant *Ocimum sanctum*** **Linn**.

**Kanika Chowdhary**1 and **Nutan Kaushik**2*

1TERI University, 10th Institutional Area, Vasant Kunj, New Delhi-110070, India

2The Energy and Resources Institute (TERI), India Habitat Center, Lodhi Road, New Delhi – 110003, India

* Corresponding author: Nutan Kaushik, TERI, IHC, Lodhi Road, New Delhi – 110003, India

Phone: 91-11-24682100/ 24682111, Fax: 91-11-24682144/24682145, Email: kaushikn@ teri.res.in

**S2 Table** Dual culture bioassay results of endophytic fungi isolated from *O. sanctum* against phytopathogens

| **S. No.** | **Code no** | **Endophytic fungi isolate** | ***S. sclerotiorum*** | ***F.oxysporum*** | ***B.cinerea*** | ***R.solani*** |
| --- | --- | --- | --- | --- | --- | --- |
| 1 | Ocl-1 | *Hypocrea sp.* | -^a^ | +^b^ | - | - |
| 2 | Ocl-12 | *Hypocrea sp.* | - | + | + | - |
| 3 | Ocl-13.1 | *Rhizopus oryzae* | - | + | - | - |
| 4 | Ocl-13.1' | *Penicillium sp.* | + | + | + | - |
| 5 | Ocl-2 | *Hypocrea sp.* | - | + | - | - |
| 6 | Ocl-2.1 | *Alternaria alternata* | - | + | - | - |
| 7 | Ocl-3.1.1 | *Hypocrea sp.* | - | + | + | - |
| 8 | Ocl-3.1.2 | *Hypocrea sp.* | - | + | - | - |
| 9 | Ocl-4.1 | *Hypocrea sp.* | - | + | - | - |
| 10 | Ocl-4.2 | *Hypocrea sp.* | - | + | - | - |
| 11 | Ocl-7.1 | *Penicillium crustosum* | - | + | + | - |
| 12 | Ocl-7.2 | *Fusarium solani* | - | + | + | - |
| 13 | Ocl-8.1 | *Rhizopus oryzae* | - | + | + | - |
| 14 | Ocl-8.1.2 | *Alternaria alternata* | - | + | + | - |
| 15 | Ocl-8.1.3 | *Alternaria alternata* | - | + | + | - |
| 16 | OSDSS-1.2 | *Chaetomium coarctatum* | - | - | + | - |
| 17 | OSDSS-1.3 | *Alternaria sp.* | + | + | - | - |
| 18 | OSDSS-1.4 | *Hypoxylon sp.* | + | + | + | - |
| 19 | OSDSS-2.5 | *Chaetomium coarctatum* | + | + | + | - |
| 20 | OSDSL-2.7 | *Alternaria sp.* | - | - | - | - |
| 21 | OSDSL-2.8 | *Chaetomium coarctatum* | - | + | + | - |
| 22 | OSDSL-3.10 | *Diaporthe phaseolorum* | + | + | + | - |
| 23 | OSDSL-3.12 | *Hypoxylon sp.* | - | - | + | - |
| 24 | OSDSL-5.12 | *Setosphaeria rostrata* | - | - | + | - |
| 25 | OSDSL-5.5 | *Setosphaeria rostrata* | - | - | + | - |
| 26 | OSDSL-5.6 | *Alternaria alternata* | + | + | - | - |
| 27 | OSDSL-6.12 | *Alternaria alternata* | + | + | + | - |
| 28 | OSDSL-7.2 | *Hypoxylon sp.* | - | - | + | - |
| 29 | OSDSL-7.4 | *Chaetomium coarctatum* | - | - | - | - |
| 30 | OSDSL-7.5 | *Chaetomium coarctatum* | - | - | - | - |
| 31 | OSDSL-8.5 | *Rhizoctonia bataticola* | + | - | - | - |
| 32 | OSDSL-9.10 | *Chaetomium coarctatum* | - | + | + | - |
| 33 | OSDSL-9.8 | *Fusarium proliferatum* | - | - | + | - |
| 34 | OSHS-1.1 | *Macrophomina phaseolina* | + | + | + | - |
| 35 | OSHS-1.2 | *Macrophomina phaseolina* | - | + | + | - |
| 36 | OSHS-1.3 | *Rhizopus oryzae* | - | - | + | - |
| 37 | OSHL-1.4 | *Setosphaeria rostrata* | - | + | - | - |
| 38 | OSHL-2.1 | *Macrophomina phaseolina* | + | + | + | - |
| 39 | OSHL-2.5 | *Meyerozyma sp.* | - | + | + | - |
| 40 | OSHL-2.6 | *Rhizopus oryzae* | + | + | - | - |
| 41 | OSHS-3.1 | *Macrophomina phaseolina* | + | + | + | - |
| 42 | OSHL-2.3 | *Fusarium proliferatum* | + | + | + | - |
| 43 | OSHL-4.1 | *Aspergillus niger* | + | - | + | - |
| 44 | OSHL-4.2 | *Sympodiomyces sp* | - | + | + | - |
| 45 | OSHL-4.4 | *Macrophomina phaseolina* | + | + | + | - |
| 46 | OSHL-4.5 | *Macrophomina phaseolina* | - | + | - | + |
| 47 | OSHL-4.6 | *Aspergillus niger* | + | + | + | - |
| 48 | OSHL-5.1 | *Macrophomina phaseolina* | + | + | - | - |
| 49 | OSHL-5.2 | *Alternaria sp.* | + | + | + | - |
| 50 | OSHSL-2.1 | *Diaporthe phaseolorum* | + | + | + | + |
| 51 | OSHSL-3.1 | *Diaporthe phaseolorum* | - | + | - | + |
| 52 | OSHSL-3.2 | *Alternaria tenuissima* | - | + | - | - |
| 53 | OSHSL-5.4 | *Fusarium solani* | + | + | - | - |
| 54 | OSHSL-7.1 | *Rhizoctonia bataticola* | + | + | - | + |
| 55 | OSHSL-7.2 | *Alternaria tenuissima* | - | + | - | - |
| 56 | OSHSL-7.3 | *Rhizoctonia bataticola* | + | + | - | - |
| 57 | OSHSL-7.4 | *Alternaria tenuissima* | - | + | - | - |
| 58 | OSHSS-1.1 | *Fusarium proliferatum* | - | + | - | - |
| 59 | OSHSS-1.3 | *Fusarium proliferatum* | + | + | + | - |
| 60 | OSHSS-1.4 | *Diaporthe phaseolorum* | + | + | - | + |
| 61 | OSHSS-2.3 | *Diaporthe phaseolorum* | + | + | - | + |
| 62 | OSHSS-3.1 | *Diaporthe phaseolorum* | + | + | + | - |
| 63 | OSHSS-3.2 | *Bipolaris maydis* | - | + | - | + |
| 64 | OSHSS-3.3 | *Diaporthe phaseolorum* | + | + | - | + |
| 65 | OSHSS-3.4 | *Bipolaris maydis* | + | + | - | - |
| 66 | OSHSS-3.5 | *Rhizoctonia bataticola* | - | + | - | + |
| 67 | OSHSS-4.2 | *Fusarium proliferatum* | - | + | - | + |
| 68 | OSHSS-4.3 | *Meyerozyma guilliermondii* | - | + | - | + |
| 69 | OSHSS-4.4 | *Rhizoctonia bataticola* | - | + | - | + |
| 70 | OSHSS-5.1 | *Fusarium proliferatum* | - | + | - | - |
| 71 | OSHSS-5.2 | *Sympodiomyces sp* | - | + | - | + |
| 72 | OSHSS-5.3 | *Rhizoctonia bataticola* | + | + | - | + |
| 73 | OSML-10.7 | *Curvularia lunata* | + | + | - | - |
| 74 | OSML-3.8 | *Penicillium sp.* | + | + | + | + |
| 75 | OSML-5.4 | *Colletotrichum sp.* | + | + | + | - |
| 76 | OSML-5.5 | *Colletotrichum sp.* | + | + | + | + |
| 77 | OSML-6.2 | *Penicillium sp.* | - | + | + | + |
| 78 | OSML-6.4 | *Penicillium sp.* | - | + | + | + |
| 79 | OSML-8.2 | *Penicillium sp.* | - | + | - | + |
| 80 | OSML-8.3 | *Aspergillus niger* | + | + | + | + |
| 81 | OSMS-1.5 | *Colletotrichum sp.* | - | + | + | + |
| 82 | OSMS-1.7 | *Colletotrichum sp.* | - | + | + | - |
| 83 | OSMS-2.2 | *Aspergillus niger* | + | + | + | - |
| 84 | OSMS-2.3 | *Meyerozyma guilliermondii* | + | + | + | + |
| 85 | OSMS-2.4 | *Aspergillus niger* | + | + | + | + |
| 86 | OSMSL-2.2 | *Aspergillus niger* | + | + | + | + |
| 87 | OSMSL-3.1 | *Aspergillus niger* | - | - | + | - |
| 88 | OSMSL-5.1 | *Aspergillus niger* | + | + | + | - |
| 89 | OSMSS-1.3 | *Aspergillus niger* | - | + | - | - |
| 90 | OSMSS-3.1 | *Fusarium verticillioides* | - | - | + | - |

“a^”^ absence of antagonistic activity against tested phytopathogen; “b“ presence of antagonistic activity against tested phytopathogen
